# Repeat Genicular Nerve Cooled Radiofrequency Ablation: Retained Efficacy or Diminishing Returns?

**DOI:** 10.3390/jcm14124194

**Published:** 2025-06-12

**Authors:** James N. Nitz, Barnabas T. Shiferaw, Michael J. Bartley, Jarod R. Moyer, Kylie K. Ruprecht, Andrew Y. Matta, Alaa Abd-Elsayed

**Affiliations:** Department of Anesthesiology, University of Wisconsin, Madison, WI 53792, USA; james.nitz@wisc.edu (J.N.N.); bshiferaw@wisc.edu (B.T.S.); mbartley2@wisc.edu (M.J.B.); jrmoyer2@wisc.edu (J.R.M.); kruprecht@wisc.edu (K.K.R.); aymatta@wisc.edu (A.Y.M.)

**Keywords:** knee pain, radiofrequency ablation, chronic pain

## Abstract

**Introduction:** Radiofrequency ablation (RFA) of the genicular nerve is an increasingly common treatment for chronic knee pain, refractory to conservative measures. However, RFA often provides significant but temporary relief, and patients return for repeat RFAs for the treatment of their pain. This study investigates the efficacy of repeat RFAs compared to the initial RFA for patients who receive repeat RFAs for chronic knee pain. **Methods:** This study is a retrospective chart review and analysis that was conducted between 2015 and 2023. Groups were determined by the number of RFA procedures that patients received, and a one-tailed ANOVA test was performed to assess the statistical significance of the initial RFA and the three repeat RFA groups. A one-way ANOVA was performed to analyze statistical differences between percent improvement, preoperative pain scores, and the duration of improvement using the visual analog scale (VAS). A *p*-value of statistical significance was set at *p* < 0.05. A paired two-tailed *T*-test was carried out, individually comparing the initial RFA to the three repeat groups for percent improvement. A paired t-test was also carried out to compare initial and first repeat RFAs for preoperative pain score and duration of improvement. **Results:** A total of 42 patients underwent repeat RFA procedures, with 8 receiving bilateral treatments, totaling 50 knees and 116 procedures. The original RFA group demonstrated a mean percent improvement of 75% ± 25% (mean ± SD) and a duration of improvement of 9.46 ± 5.45 months. The first repeat group had a mean percent improvement of 66% ± 29% and a duration of improvement of 8.77 ± 7.32 months. The second repeat group had a mean percent improvement of 67% ± 24% and a duration of improvement of 10.00 ± 2.45 months. The third repeat group had a mean percent improvement of 85% ± 20% and a duration of improvement of 4.00 months. ANOVA revealed no statistically significant differences among the groups in preoperative scores (*p* = 0.40), percent improvement (*p* = 0.25), or duration of improvement (*p* = 0.79). Paired t-tests showed a significant decrease in percent improvement in the first repeat RFA compared to the original RFA (*p* = 0.04), but no significant differences were observed in preoperative scores (*p* = 0.057) or duration of improvement (*p* = 0.175). No significant differences were found in percent improvement via paired *T*-test between the original RFA and the second (*p* = 0.75) or third repeats (*p* = 0.21). **Conclusions**: The repeat RFA of genicular nerves retains a clinically significant level of pain reduction for chronic knee pain. However, this study demonstrated decreased pain relief following the first repeat RFA compared to the initial RFA when analyzing individual knees sequentially via a paired *T*-test. An analysis of initial, first, second, and third repeat groups via ANOVA showed no difference in percent improvement, duration of pain relief, or preoperative pain scores.

## 1. Introduction

Chronic knee pain is a common and debilitating condition, significantly impacting patients’ quality of life, with chronic knee pain being reported by over 650 million people worldwide [1,2]. Chronic knee pain is often caused by osteoarthritis, but other common causes include persistent post-surgical pain, meniscus tears, and rheumatoid arthritis [1,3].

Standard treatment options for chronic knee pain include physical therapy, weight loss, analgesics, anticonvulsants, opioids, steroid injections, and other procedural interventions up to and including total knee replacement [4]. Among the available treatment options, radiofrequency ablation (RFA) has emerged as a promising minimally invasive technique for managing chronic knee pain, particularly in patients who are poor surgical candidates or who wish to delay more invasive procedures such as total knee replacement [5,6,7,8]. RFA involves the insertion of a needle probe under ultrasound or fluoroscopic guidance to target the nerve that is believed to be responsible for the patient’s source of pain. Once in place, radiofrequency energy is transmitted through the probe to create a lesion on the nerve tissue, disrupting afferent pain signals. Cooled radiofrequency ablation (c-RFA) is performed with a water-cooled tip, which is in contrast with non-cooled thermal techniques [8].

Although RFA has been shown to provide pain relief, its benefits are typically temporary, with the resumption of pain occurring as nerve regeneration occurs. The duration of pain relief following RFA is highly variable, with patients commonly experiencing a return of pain from 6 to 24 months following RFA [5,9,10,11]. Given the transient nature of relief, many patients return for repeat RFA procedures to manage recurring pain.

Despite the growing popularity of RFA for treating knee pain and the concurrent use of repeated RFA to manage returning pain, data on the efficacy of repeated RFA procedures remain limited. This retrospective study investigates a cohort of patients who underwent multiple RFA procedures of the genicular nerves to treat chronic knee pain. We aim to compare the effectiveness of the initial ablation with subsequent ablations, providing insights into the reproducibility of this therapeutic approach.

## 2. Methods

### 2.1. Patient Selection and Chart Review

This study is a single-center, retrospective review of the electronic health records at the University of Wisconsin Hospitals and Clinics between 2015 and 2023, as well as a subsequent analysis of data from patients who received c-RFA of the genicular nerves as a treatment for chronic knee pain. Only those who received c-RFA of the genicular nerves and at least one repeat RFA procedure on the same knee between 2015 and 2023 were included in the study. The primary goal of the study was to determine if repeated RFA procedures of the genicular nerves retained their efficacy for reducing chronic knee pain as compared to initial RFA procedures, which are shown to be an effective modality for treating knee pain.

Patients were analyzed in four groups based on how many RFA procedures they received on a given knee. The original RFA group contains the result from each patient’s first RFA procedure; all patients included underwent at least one repeat RFA. The first repeat group includes the results of each patient’s second RFA, following an initial RFA. The second repeat group contains results from those who underwent a second repeat RFA following one original RFA and one repeat RFA. The third repeat group contains results from those who received a third RFA following one original RFA and two repeat RFAs.

Comprehensive data collection included demographics such as age, sex, body mass index (BMI), and relevant past medical history. Other data collected included pre- and post-ablation pain scores, the patient-reported percent pain relief, and duration of relief.

### 2.2. End Points

The primary endpoint of this study was the percent improvement in pain following RFA, defined as a relative reduction in patient-reported pain scores from preoperative to postoperative assessments. This measure was used to evaluate and compare the efficacy of initial and repeat RFAs. Secondary endpoints included preoperative pain scores and duration of pain relief, which was defined as the number of months patients reported sustained improvement after each RFA procedure.

### 2.3. Statistical Analysis

Data were grouped by number of repeat RFAs, into groups of singular RFA, first repeated RFA, second repeat RFAs, and third repeat RFAs. All data were collected and organized using Microsoft Excel spreadsheets (Version 16.96.1), and a one-tailed ANOVA test was performed to assess the statistical significance between the initial RFA and the three repeat RFAs group. A one-way ANOVA was performed to analyze statistical differences between percent improvement, preoperative pain score, and duration of improvement. A *p*-value of statistical significance was set at *p* < 0.05. A paired two-tailed *T*-test was carried out, individually comparing the initial RFA to the three repeat groups for percent improvement. A paired *t*-test was also carried out to compare the initial to the first, second, and third repeat RFA groups, respectively, for preoperative pain score and duration of improvement. Each knee (right or left knee of a particular patient) represented one unit in all statistical analyses. Data were tested for normality using a Kolmogorov–Smirnov test at an alpha level of 0.05.

### 2.4. Pain Scoring

The visual analog scale (VAS), a widely adopted method of measuring patients’ pain intensity and any changes, was used to assess the pain scores of patients in this study. If patient charts included VAS pain scores, but a percentage of pain improvement was not reported, the percentage improvement was calculated using the pre- and post-ablation scores (e.g., a pre-ablation VAS pain score of 10/10 and a post-ablation score of 5/10 indicated a 50% improvement). If patient charts included a pre-ablation pain score and percentage improvement, but a post-ablation pain score was not reported, the post-ablation pain score was derived from the given data (e.g., a preoperative pain score of 10/10 with a 25% improvement indicated a postoperative pain score of 7.5/10). Patient charts that reported the complete resolution of pain were reported as a 100% pain improvement. Patient charts that reported no difference in pre- and post-ablation pain scores were reported as 0% pain improvement. If a range of pain scores was reported, the highest and lowest numbers were averaged. Post-ablation pain scores were excluded from analysis if they followed another therapy or procedure for knee pain, such as injections or total knee arthroplasty. If the duration of improvement was not clearly stated in a follow-up appointment, it was calculated from the time of the procedure to the first mention of a significant return of symptoms in the patient chart. For the patients in the repeat groups, a similar analysis was used. If the duration of pain relief was not clearly stated in the chart, it was calculated from the time of the repeat procedure to the first mention of the return of symptoms in the patient chart.

### 2.5. Diagnostic Block

Before proceeding with radiofrequency ablation, patients were required to undergo a diagnostic block of the genicular nerves. For this, 1% lidocaine was used for subcutaneous anesthesia followed by the fluoroscopy-guided insertion of a 22-gauge 2.5-inch spinal needle into the superior lateral genicular nerve, the superior medial genicular nerve, and the inferior medial genicular nerve for the administration of 0.5–1.5 mL of 0.25% Bupivacaine or 1% lidocaine at each nerve site. A greater-than-50% reduction in pain score had to be reported from the diagnostic nerve block before moving forward to radiofrequency ablation.

### 2.6. RFA Procedure

Lidocaine (1%) was given via a 25-gauge needle for subcutaneous anesthesia. Under image-intensifier control, 17-gauge 50 mm cooled radiofrequency probes with 4 mm active tips were inserted at the junction of the medial femoral shaft with the femoral epicondyle for the superior medial genicular nerve, the junction of the medial tibial shaft and tibial epicondyle for the inferomedial genicular nerve, and the junction of the lateral femoral shaft and femoral epicondyle for the superolateral genicular nerve. The probes were advanced using tunnel technique until bony contact was obtained. Probe tip locations were then determined with lateral-view fluoroscopy to confirm bony contact. Motor testing was then performed at 2 Hz to ensure no motor stimulation. In total, 0.5–1.5 mL of 2% lidocaine and/or 0.25% bupivacaine was injected at each site before RF lesioning. RF lesioning was set at 80 degrees Celsius for 2 min and 30 s.

### 2.7. Follow-Up

Patients were scheduled for a six-week follow-up appointment after the RFA procedure, at which point pain diaries were uploaded to the chart when available. For those without pain diaries or a six-week follow-up, pain scores were obtained from the first postoperative appointment with any specialty, provided that no interval alternative therapy had been initiated to treat knee pain. Some were lost to follow-up until pain resumed at which point, they were seen again in the pain clinic.

Following the resumption of pain, some patients select to undergo repeat RFA. Repeat RFA on the genicular nerve was offered to all appropriate patients with ongoing knee pain who were being following in the pain clinic; however, decisions were ultimately made by the patient.

### 2.8. Consent and IRB

The University of Wisconsin Institutional Review Board reviewed and exempted this study. Compliance with patient confidentiality was kept throughout the study.

## 3. Results

A total of 521 RFA procedures were reviewed. Patients were included if they underwent a cooled RFA procedure with recorded preoperative and postoperative pain scores in the chart. Patients were included only if they underwent at least two RFA procedures on the genicular nerve for knee pain on the same knee. A total of 42 patients and 116 procedures were included in the review. The duration of pain relief was calculated according to the time from the procedure to the significant recurrence of pain, as recorded in the chart. Patients reporting no duration of relief were excluded from the analysis of the duration of relief but were included in the analysis of degree pain relief and preoperative pain scores. The duration of relief was found for a total of 75 procedures—42 procedures for initial RFA, 26 for first repeat RFA, 6 for second repeat RFA, and 1 for third repeat RFA.

In total, 42 patients in this study underwent repeat c-RFA procedures (29 female, 13 male). A total of eight of these patients received bilateral repeat c-RFA procedures, accounting for 50 knees included in this study. In total, 38 of the knees underwent one repeat, 8 underwent two repeats, and 4 underwent three repeats, accounting for a total of 116 procedures. In the initial RFA group, the duration of relief was found for 42 of the procedures. For the first repeat group, the duration of relief was found for 26 of the procedures. For the second repeat group, the duration of relief was found for six of the procedures. For the third repeat group, the duration of relief was found for one of the procedures. The average age of the cohort was 58.3 +/− 13.4 (range 21–90) years, with a BMI of 34.91 +/− 9.33 (Table 1). The etiology of underlying knee pain included osteoarthritis, pain following knee surgery, and pain secondary to soft tissue injuries such as ligamental tears, chondromalacia, and idiopathy.

The original RFA group had a mean pre-procedure pain score of 6.48 +/− 1.69, a mean post-procedure pain score of 1.61 +/− 1.63, a mean percent improvement of 75% +/− (SD) 25%, and a mean duration of improvement of 9.46 +/− 5.45 months. The first repeat group had a mean pre-procedure pain score of 6.05 +/− 1.76, a mean post-procedure pain score of 2.20 +/− 2.06, an average percent improvement of 66% +/− 29%, and an average duration of improvement of 8.77 +/− 7.32 months. The second repeat group had a mean pre-procedure pain score of 6.83 +/− 1.32, a mean post-procedure pain score of 2.28 +/− 1.94, an average percent improvement of 67% +/− 24%, and an average duration of improvement of 10.00 +/− 2.45 months. The third repeat group had a mean pre-procedure pain score of 6.63 +/− 0.75, a mean post-procedure pain score of 1.00 +/− 1.41, an average percent improvement of 85% +/− 20%, and an average duration of improvement of 4.00 months (Table 2).

An analysis of variance (ANOVA) was used to compare the results of the original RFA procedure to those of the three repeat groups. The results of the ANOVA did not demonstrate statistically significant differences in average preoperative scores (*p* = 0.40), average percent improvement (*p* = 0.25), or the average duration of their improvement (*p* = 0.79) (Table 3).

Paired *T*-tests were also performed to look at each group and separately compare them to the original RFA. A statistically significant decrease in average percent improvement was found in the first repeat RFA compared to the original RFA procedure (*p* = 0.04). The average VAS percent improvement for the original RFA procedure was 75% +/− 25%, whereas that of the first repeat RFA was 66% +/− 29% (Table 2). There was no significant difference between these groups in their mean duration of improvement (*p* = 0.175) nor their average preoperative scores (*p* = 0.057). Similarly, there were no statistically significant differences found when comparing the initial RFA procedure and the second repeat for percent improvement of pain (*p* = 0.75), preoperative scores (*p* = 0.38), or duration of improvement (*p* = 0.55). No statistically significant differences were found when comparing the initial RFA and the third repeat for percent improvement (*p* = 0.21) or preoperative scores (*p* = 0.06). The duration of improvement could not be compared between the initial and third repeat group due to the lack of data points in the third repeat group (Table 4).

## 4. Discussion

### 4.1. Summary of Results

The results of this study demonstrate an interesting phenomenon. The ANOVA showed no significant differences between the original RFA and the three repeat groups, indicating that repeat RFA procedures retain their efficacy. According to the ANOVA analysis, there is no difference between the efficacy of an initial RFA procedure and the first, second, or third repeat RFA. However, when each knee was compared to the initial RFA procedure and first repeat RFA, a statistically significant decrease in pain reduction was noted for the repeat RFA compared to the original RFA (Table 4). Therefore, each knee should expect a reduced efficacy with their first repeat RFA. While there is a decreased level of pain reduction between initial RFA and first repeat RFA, there is still a high level of pain reduction, indicating that a substantial and clinically significant amount of pain relief will still likely occur (Table 2).

There were no statistical differences between the durations of relief according to either ANOVA or paired *T*-test. Therefore, although the amount of pain reduction may decrease in subsequent RFAs on the same knee, the duration of relief is not affected. Paired *T*-tests also demonstrated no significant difference in pain reduction between the initial and the second repeat RFA procedure or between the initial and the third repeat RFA procedure. The data in this study suggest that serial repeat procedures may not be associated with decreased efficacy. In addition, they are associated with clinically meaningful pain reduction, as indicated by an average percent pain improvement of 66%, 67%, and 85% for the first, second, and third RFA repeats, respectively, (Table 2).

### 4.2. Physiologic Mechanisms for Return of Pain

While there have been reports of permanent pain reduction following RFA, it is considered a temporary pain relief method, with the return of pain occurring within a year for the majority of patients [5]. Pain return in patients undergoing RFA is likely multifactorial, with the primary mechanisms revolving around nerve regeneration [11]. During radiofrequency ablation, the continuity of axons is interrupted using thermal energy, and the nerve undergoes Wallerian degeneration. During this interruption, there is an injury to the myelin, axon, and endoneurium; however, it largely spares the disruption of the fascicular arrangement, perineurium, and epineurium. This degree of nerve injury is classified as third-degree axonotmesis and is subject to the regeneration of the nerve [12]. Aside from frank nerve regeneration, the sensory nerve regains function postoperatively through remyelination and the collateral sprouting of preserved axons. The degree of frank regeneration compared to sprouting and remyelination depends on the degree of injury to the nerve. This is likely a leading reason for the wide variety of durations of pain relief depending on the precision and extent of neurolysis during the ablation [12].

This has been seen in studies comparing the duration of pain relief between chemical ablation and radiofrequency ablation. One study investigating the duration of relief of chemical (alcohol) ablation demonstrated a relief time of 24.0 months compared to 10.7 months with thermal radiofrequency ablation. This significant increase in the duration of relief is likely attributable to a larger lesion being ablated, as well as a higher degree of neurolysis being available through chemical means [13]. However, chemical ablation is associated with increased serious adverse effects, including skin necrosis, damage to non-targeted tissue, prolonged motor paralysis, and anesthesia dolorosa. For this reason, it is not used in everyday clinical practice and is generally reserved for terminal cancer patients with less than one year of life expectancy [14].

Currently, no definitive mechanisms are described in the literature for why repeat RFAs may have a lower efficacy than their initial counterparts. However, mechanisms could include fibrosis, in which anatomic changes hinder nerve targeting, or changes in tissue heat conduction, which may result in a lower degree of neurolysis. Nerve regeneration from the damage caused in the previous RFAs may result in aberrant or disorganized nerve fibers that provide a less-simple target for ablation. Nerve sprouting from collateral nerves not targeted by typical ablations may represent another mechanism for resistance on repeat RFAs. In knee pain specifically, RFAs may provide limited relief due to the sensory fibers of articular branches of the tibial, saphenous, femoral, and common peroneal nerves [15]. More research is needed to investigate these mechanisms further.

### 4.3. Comparison to the Current Literature

Repeat RFA outcomes have been studied at various nerves and anatomic locations, and successful pain relief and duration of pain relief remain consistent in repeat RFAs. The findings of this study appear consistent among various nerves targeted for RFA [16,17,18,19,20]. However, the specific duration of relief varies depending on the nerve of interest. Currently, there are no available studies focusing on repeated RFA procedures targeting the genicular nerves; however, the data in the present study correlate well with the results of articles focusing on repeat RFAs on other more commonly studied nerves [16,17,18,19,20].

Anecdotally, physicians have noted that the intensity of pain can be worse following the resumption of pain compared to their original level of pain before any RFA procedure. In addition, increased pain is one of the adverse effects reported following RFA [5]. However, this phenomenon was not observed in this study. A paired *T*-test evaluating the preoperative pain scores before the initial genicular nerve RFA and the first repeat RFA showed no difference (Table 4). In addition, the ANOVA test showed no significant difference in preoperative pain scores between all groups (Table 3).

### 4.4. Clinical Implications

Clinical practice and patient recommendations can benefit from the findings of this study. While the results of the present study and past investigations indicate high success rates in repeat RFA procedures, managing patient expectations about the extent and duration of pain relief is essential for providers to communicate with patients. Patients may be counseled that they may receive less pain relief than their initial RFA. However, they should still be offered the repeat RFA upon the return of pain because of the clinically significant level of pain relief conferred by repeat RFAs. Patients should expect to receive a similar duration of improvement as their initial RFA. Lastly, once pain returns, it will likely not be significantly different from the baseline pain experienced before their first RFA.

### 4.5. Limitations

The statistical tests, including ANOVA and t-tests, that were chosen to analyze the data in this study were chosen for their relative familiarity in the scientific community and their suitability with relatively small sample sizes. However, these tests present limitations including a lack of correction of multiple comparisons and less accountancy of covariates. Data interpretation should consider these limitations.

This retrospective study is subject to selection bias. Patients who responded positively to their initial RFA may be more likely to pursue repeat RFAs for subsequent pain relief. This is reflected in the data, as the percent improvement was higher than the averages found in a previous study on the same patient population that included non-repeats [5]. Ultimately, this bias is likely less significant from a clinical perspective because the study population is representative of patients who would likely choose to undergo repeat RFAs. Further investigation is required to determine the potential for success of a repeat RFA procedure after a failed initial RFA.

Additionally, postoperative pain scores were analyzed using the visual analog scale. However, the recent literature has shown the benefit of functional assessment tools as a more comprehensive metric for the efficacy of a procedure and is an inherent limitation of this retrospective study.

This study’s retrospective, single-center nature constitutes other potential limitations. Pain scores are subjective and may vary from patient to patient. This was primarily accounted for by using percent improvement as the basis of our statistical analysis. Data focusing on functional outcomes rather than pain scores are becoming the gold standard in pain research and would be valuable in future investigations.

This was a single-center study. However, procedures were carried out by a variety of physicians, introducing variability in technical approaches. The study has a small sample size, decreasing statistical power, particularly in the second and third repeat categories. Research with larger sample sizes is needed to determine if serial repeat RFAs retain their efficacy. Furthermore, the current study exclusively utilized cooled RFA procedures, which may impact the comparison of repeat RFA to other nerve ablation modalities.

Due to the multivariable nature of chronic knee pain and the low sample size, this paper did not analyze patient groups according to the underlying etiology of knee pain. Future research needs to be conducted to evaluate whether the underlying etiology of knee pain affects the efficacy of repeated RFAs.

Lastly, due to a loss of follow-up and the limitations of chart review being excluded from the review for patients for whom no postoperative pain scores were found, this may result in possible attrition bias. This is especially true for the analysis of duration of improvement, in which many patients were excluded due to loss of follow-up or the uncertainty of the precise return of symptoms.

## 5. Conclusions

Repeat radiofrequency ablation of the genicular nerves still offers significant improvement for patients. However, this study demonstrated decreased pain relief following the first repeat RFA compared to the initial RFA when analyzing individual knees in a sequential manner. When examined according to group, there were no significant differences in the reduction in pain among the initial RFA, first repeat RFA, second repeat RFA, or third repeat RFA groups. There were no significant differences regarding pre-procedural pain scores or the duration of relief when comparing initial RFAs to repeat RFA groups. Similarly, when analyzing individual knees compared to each other, there were no significant differences found between initial, first, second, and third repeat RFAs for preoperative pain scores or duration of relief. Overall, the data from this study show that repeated RFA of the genicular nerves retains clinical efficacy in treating chronic knee pain.

## Figures and Tables

**Table 1:** Patient demographics.

Total c-RFA (n)	116
Total patients (n)	42
Total knees (n)	50
Sex (F/M)	29/13
Sex of patient for each procedure (F/M)	80/36
Age (years), mean +/− SD	58.3 +/− 13.4
BMI (kg/m^2^), mean +/− SD	34.91 +/− 9.33

BMI = body mass index; c-RFA = cooled radiofrequency ablation.

**Table 2:** Original and repeat RFA pain scores, percent improvement, and duration of improvement.

	Original RFA (n = 50)	First Repeat (n = 50)	Second Repeat (n = 12)	Third Repeat (n = 4)	All Repeats (n = 66)
VAS pain score pre-procedure	6.48 ± 1.69	6.05 ± 1.76	6.83 ± 1.32	6.63 ± 0.75	6.26 ± 1.64
VAS pain score post-procedure	1.61 ± 1.63	2.20 ± 2.06	2.28 ± 1.94	1.00 ± 1.41	2.25 ± 2.03
VAS % improvement	75% ± 25%	66% ± 29%	67% ± 24%	85% ± 20%	66% ± 28%
	Original RFA (n = 42)	First Repeat (n = 26)	Second Repeat (n = 6)	Third Repeat (n = 1)	All Repeats (n = 33)
Duration of Improvement (months)	9.46 +/− 5.45	8.77 +/− 7.32	10.00 +/− 2.45	4.00	8.84 +/− 6.66

Data are presented as mean ± SD. The total number is provided as the n value. VAS = visual analog scale.

**Table 3:** Summary of ANOVA.

	*df*	*F*	Significance
VAS pain score pre-procedure	112	1.00	0.40
VAS % improvement	112	1.38	0.25
Duration of Improvement	112	0.35	0.79

df = degrees of freedom. Significance is defined as *p* < 0.05. VAS = visual analog scale.

**Table 4:** Summary of paired *T*-testing.

	Original vs. First Repeat	Original vs. Second Repeat	Original vs. Third Repeat
VAS pain score pre-procedure	*p* = 0.06	*p* = 0.38	*p* = 0.55
VAS % improvement	*p* = 0.04	*p* = 0.75	*p* = 0.21
Duration of Improvement	*p* = 0.17	*p* = 0.06	NA

Significance is defined as *p* < 0.05. VAS = visual analog scale.

## Data Availability

All data was stored securely in accordance with University of Wisconsin policies. Due to institutional restrictions designed to protect the most secure handling of patient data, the study data are not available publicly.
